# Experience of Secondary Abdominal Pregnancy Reported at a Rural Tertiary Care Center of Western India: A Case Series

**DOI:** 10.7759/cureus.55663

**Published:** 2024-03-06

**Authors:** Ritesh R Joshi

**Affiliations:** 1 Obstetrics and Gynaecology, Pramukhswami Medical College, Anand, IND

**Keywords:** obstetrics ultrasound, hystrectomy, tubal recanalization, advanced abdominal pregnancy, secondary abdominal pregnancy, ectopic pregnancy

## Abstract

Abdominal pregnancy remains a rare entity among ectopic pregnancies overall; however, it carries the highest risk of mortality and morbidity for the mother and the fetus. Prompt diagnosis and early intervention remain the main modality of treatment to prevent catastrophic complications because of abdominal pregnancy. However, many barriers exist, leading to delayed diagnosis and management. We present three cases of secondary abdominal pregnancy with different outcomes over three years from 2018 to 2021. The clinical presentation, evaluation, management plan, and outcomes of the cases are discussed separately.

## Introduction

Abdominal pregnancy is rare and remains an enigma for treating obstetricians in their day-to-day practice. In abdominal pregnancy, the implantation of the fertilized ovum occurs outside the reproductive tract, but potentially anywhere else in the peritoneal cavity [1]. Overall incidence of abdominal pregnancy varies in the population, mainly because of associated cofactors such as geographic location, low socioeconomic status, and conditions that directly or indirectly affect the anatomical and physiological function of fallopian tubes [2].

Although an ectopic pregnancy is typically fatal, an abdominal pregnancy is unusual in that it may result in a term successful outcome [3]. Maternal mortality ranges from one in 10 to one in 200 patients, while perinatal mortality varies, with an average of one out of two neonates [4]. Advanced abdominal pregnancy is defined as a gestational age of >20 weeks at presentation [5], with further subdivision as primary or secondary abdominal pregnancy. In primary abdominal pregnancy, the fertilized ovum is implanted directly into the peritoneal cavity; however, if the initial implantation of the ovum occurs in fallopian tubes and tubal abortion or rupture occurs, the ovum can reimplant in the peritoneal cavity as a secondary abdominal pregnancy [6]. This latter type of abdominal pregnancy is the most common [6].

Patients with abdominal pregnancy are usually asymptomatic or have nonspecific signs and symptoms [7]. Therefore, diagnosis poses a challenge for the obstetrician and the radiologist. Ultrasonography remains the main modality for diagnosis, and if done in the first trimester, it has more sensitivity and specificity than in later trimesters, although diagnostic error confounds to be 50%-90% mainly because of inexperienced sonologists [8].

The outcome of abdominal pregnancy depends on various factors, such as gestational age at presentation, site of implantation, and overall general health of the mother. Hence, early diagnosis and intervention are critical for success. We present a case series of three secondary abdominal pregnancies reported at our tertiary care center with varied presentations and outcomes.

## Case presentation

Case 1

A 30-year-old woman in her second pregnancy presented to our emergency department at 36.5 weeks of gestation. She was referred from a private hospital to our setting as her ultrasound suggested abdominal pregnancy or rudimentary horn pregnancy. The patient had a history of tubal ligation done seven years earlier, followed by a recanalization surgery around one year ago. She had no other significant medical history.

Ultrasonography was performed twice during her ongoing pregnancy, and the latest scan, without available images, aroused concerns regarding either abdominal pregnancy or a pregnancy involving a rudimentary horn. On a general examination, her vital signs were within normal limits. Abdominal examination was inconclusive.

**Figure 1:**
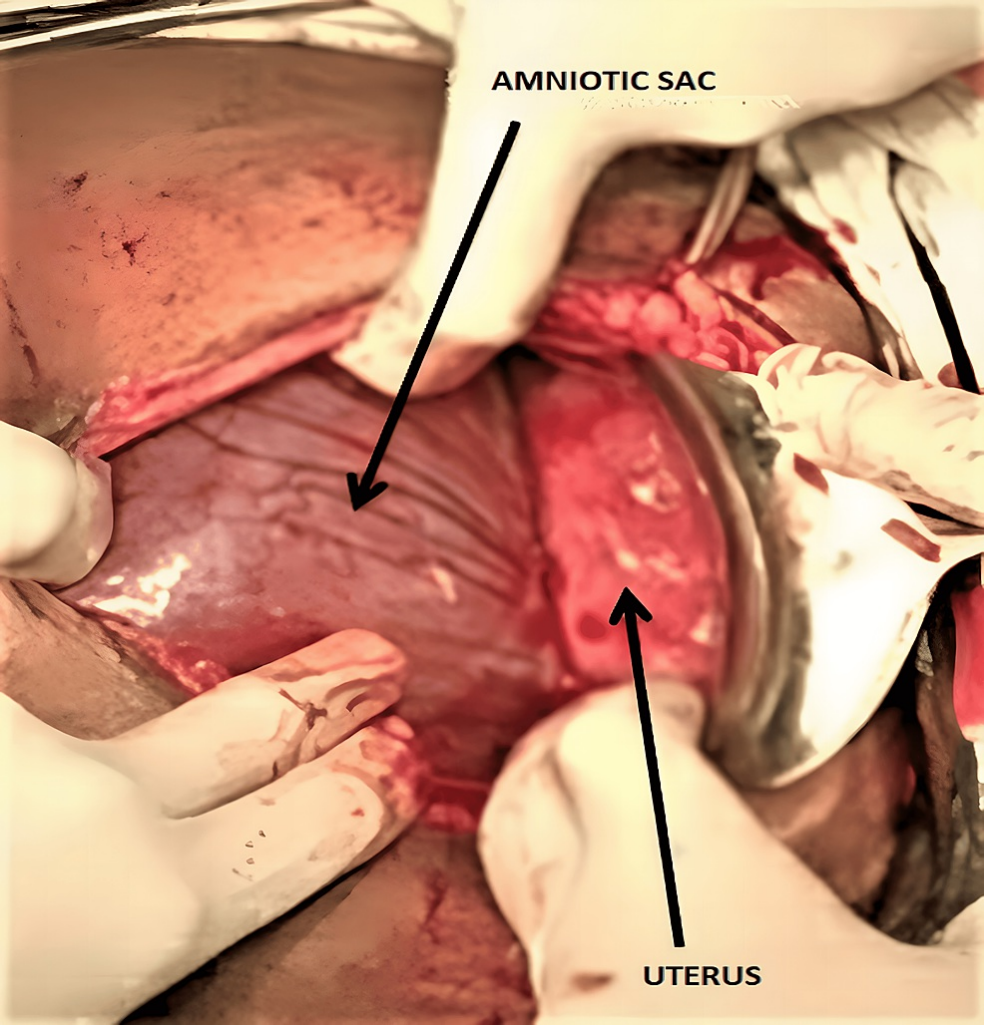
Following incision, an intact sac and uterus were seen.

An emergency laparotomy was performed because of the suspicion of an abdominal pregnancy. The fetus was seen in an intact amniotic sac, and there was no hemoperitoneum (Figure 1). A live female neonate was delivered weighing 2.5 kg. The placenta was strongly adherent to the right adnexa, ovary, and fallopian tube. The uterus and left adnexa were normal. After the delivery of the healthy newborn, a sudden gush of fresh bleeding occurred from the placental bed. A subtotal hysterectomy was performed, and hemostasis was secured. The total estimated intraoperative blood loss was 1,500 mL, and the patient was transfused with three units of cross-matched blood during the intra- and postoperative period. Both the mother and the baby progressed well and were discharged with close follow-up.

Case 2

A 24-year-old woman in her second pregnancy and with six months of amenorrhea, presented to our emergency department because of abdominal pain and per vaginum spotting for the past two days. The patient was referred from a private hospital for further management of cesarean scar pregnancy or a ruptured uterus. She had a history of cesarean section in her previous pregnancy. The patient had not received antenatal care.

On examination, the patient was hemodynamically unstable and pale, and she had cold, clammy extremities. Per abdomen examination, a tense and tender abdomen was revealed, and the uterine contour could not be identified. Laboratory tests revealed that the hemoglobin was 4 g/dL, while other parameters were within normal ranges.

**Figure 2:**
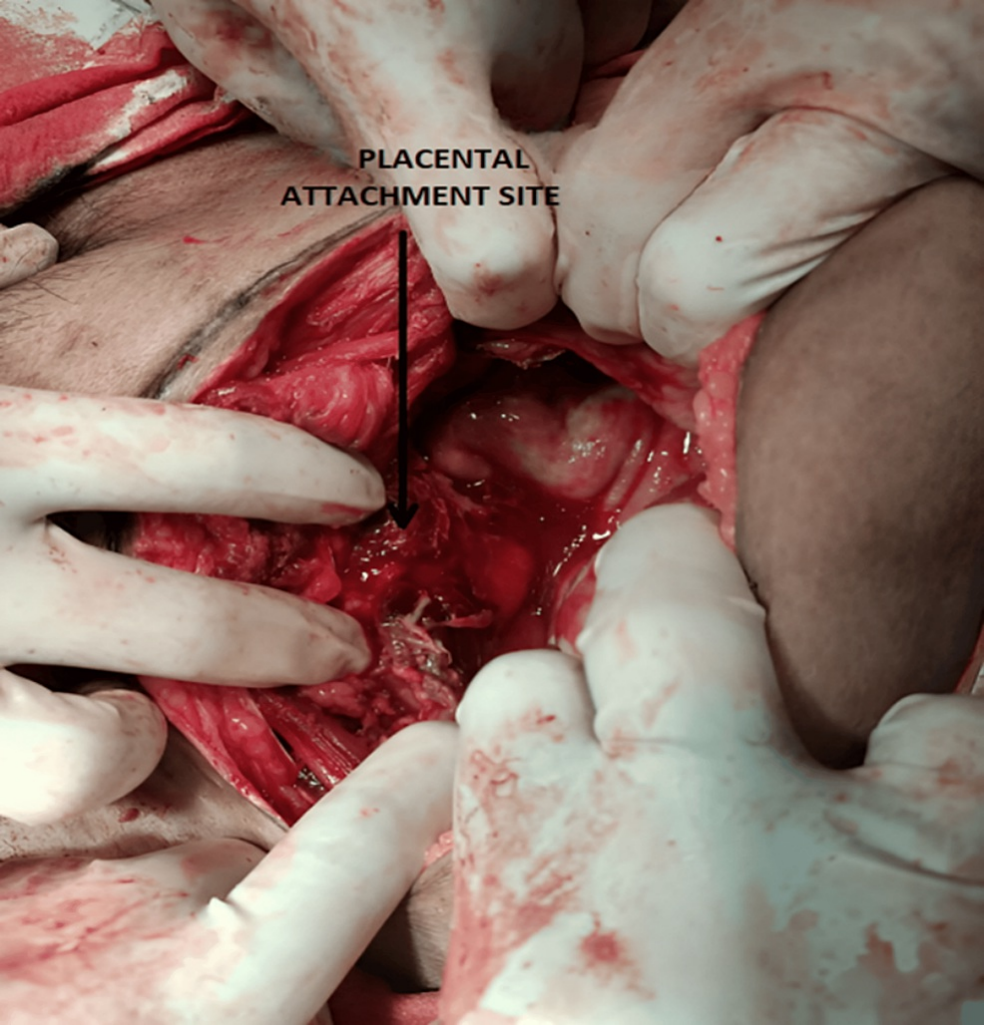
The area in the pouch of Douglas, where the abdominal pregnancy was attached.

An emergency laparotomy was done, and an old macerated female fetus was delivered from the abdominal cavity. The placenta was adherent to the right iliac fossa and removed by sharp and blunt dissection (Figure 2). Massive blood transfusion protocol was implemented intraoperatively due to hypovolemic shock. The postoperative period was uneventful. After debriefing her about the condition and its future impact, the patient was discharged with close follow-up.

Case 3

A 40-year-old grand multiparous woman was referred to our emergency department for the management of severe anemia and septic shock. She was in her fifth pregnancy, with five months of amenorrhea, and her previous pregnancies were uneventful. She did not attend antenatal checkups, thus no booking visit (initial antenatal visit) or dating scan had been done for her.

Examination revealed the following measurements: blood pressure, 100/60 mm Hg; pulse, 140 beats/min; and respiratory rate, 32 breaths/min. Her oxygen saturation was 95% on a 15 L non-rebreather mask. Based on these results, the patient’s modified early obstetrics warning score was calculated to be eight. Her blood tests revealed a hemoglobin of 4.5 g/dL, a total leukocyte count of 48,000/mm3, and a platelet count of 5,000/mm3. These test reports created a dilemma in diagnosis.

**Figure 3:**
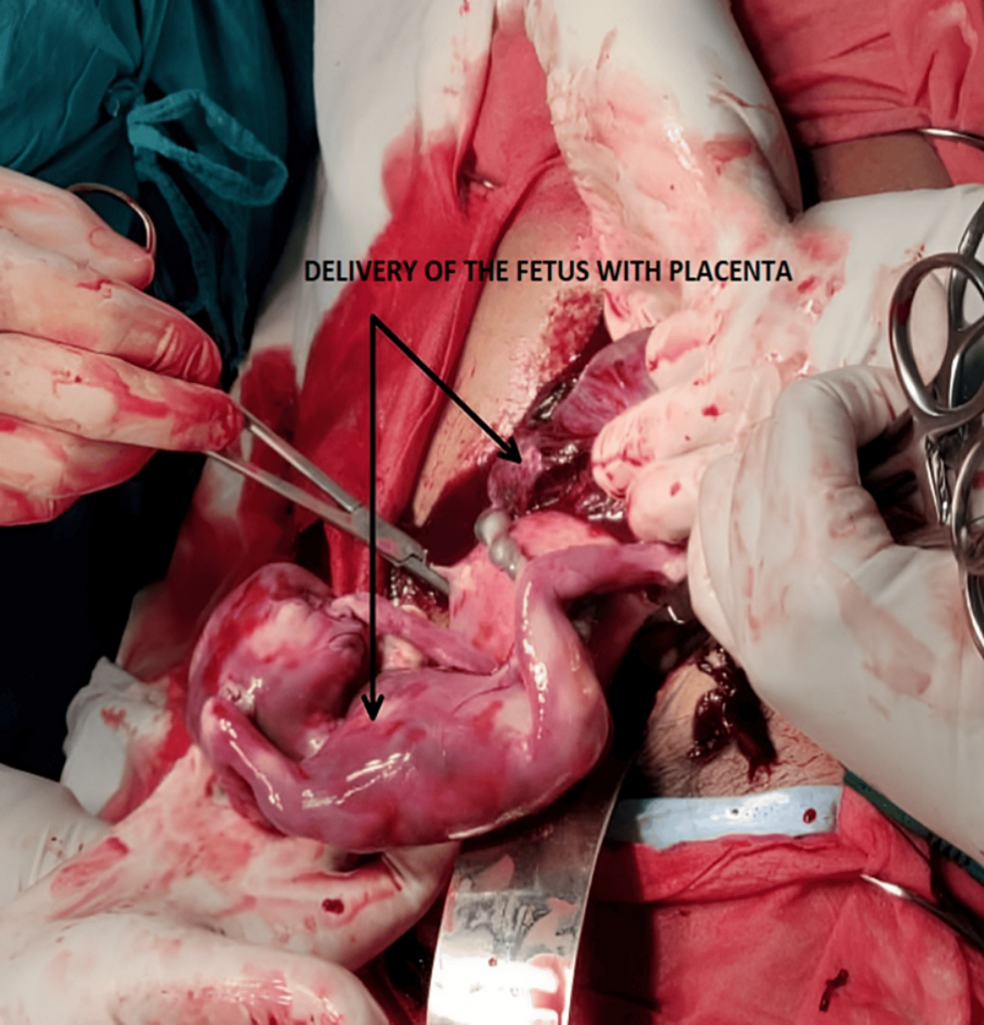
Intraoperative fetus and separated placenta were appreciated.

The massive blood transfusion protocol was activated, and as the patient’s condition deteriorated further, she was intubated, inotropes were started, and an emergency laparotomy was done. An intact sac with a fetus was observed on the left side of the uterus, attached to the back of the uterus, bowel, and omentum; approximately 700 mL of hemoperitoneum was drained out. Torrential bleeding occurred from the placental bed, and to secure hemostasis, the fetus and placenta were delivered (Figure 3), followed by a subtotal hysterectomy. Even after the transfusion of four units of packed red blood cells, four units of fresh frozen plasma, four units of platelets, and 10 units of cryoprecipitates, the patient’s vital signs worsened. She went into bradycardia and experienced cardiac arrest during the procedure; cardiopulmonary-cerebral resuscitation was started according to the advanced cardiovascular life support protocol. After 30 min of the resuscitation, we were unable to prevent her death.

## Discussion

Women with abdominal pregnancy often present with various nonspecific signs and symptoms such as pain in the abdomen, painful fetal movements, vaginal bleeding, and syncope [9]. In our case series, the first patient was asymptomatic with normal observations, while the other two patients had nonspecific complaints, including both abdominal pain and hypovolemic shock.

Ectopic pregnancy, including abdominal pregnancy, is associated with risk factors including history of previous ectopic or abdominal pregnancy, microsurgery on the tubes, pelvic inflammatory disease, and the use of intrauterine devices [10]. The first patient had a history of tubal recanalization surgery, which strongly suggests the cause for developing abdominal pregnancy in this case. However, the other two patients did not have any risk factors that could be directly or indirectly correlated with abdominal pregnancy.

Because abdominal pregnancy is a rare condition, early detection by clinical examinations or sonography is not an easy task for the modern clinician, mainly because of a lack of experience or awareness of the condition [11]. This pattern was evident in all three of our cases, with a preoperative diagnosis of abdominal pregnancy being possible in the first case because of an ultrasound, which remains the gold standard for diagnosing abdominal pregnancy [12]. However, ultrasonography by a sonologist was inconclusive in the second and third cases, and abdominal pregnancy was only confirmed during surgery.

Early detection and termination of pregnancy, either by medical or surgical methods, is usually recommended for early abdominal pregnancy [13]. However, which method to use depends on various factors, such as the gestational age, hemodynamic condition of the mother, location of the placenta, and expertise of the clinician in handling this rare entity [14].

In our series, all three patients were managed surgically because of advanced gestation age and/or compromised general condition of the mother. Whether to deliver the placenta or not mainly depends on the location and bleeding from the placental bed. In this series, the placenta was delivered intraoperatively, as two of the patients had torrential bleeding from the placental attachment. In the third patient, the placenta was partially separated from the bed, and there was no active bleeding from it.

Saving both the mother and the child remains the utmost priority for a treating doctor, but it is not possible in some cases. Our case series included both positive and negative outcomes. Both the mother and baby were saved in the first case. However, it was not possible to save the fetus in the second and third cases, and the mother did not survive in the third case. This patient’s death was because of late presentation and delay in diagnosis, and it could have been prevented by early referral and intervention.

## Conclusions

The presented case series illustrates the intricate nature of abdominal pregnancies, highlighting diverse outcomes among patients with varying gestational ages and clinical presentations. The challenges encountered in diagnosing this condition, evidenced by the varied signs and symptoms observed, emphasize the complexity and potential difficulties in early identification. This series serves as a poignant reminder to obstetricians of the indispensable role of viability scans in the early detection and management of abdominal pregnancies. By sharing our experiences, we aim to underscore the significance of vigilant monitoring and diagnostic tools in averting such tragic outcomes and, ultimately, improving care and outcomes for future cases.
